# Facial morphometrics of children with NON-syndromic orofacial clefts in Tanzania

**DOI:** 10.1186/1472-6831-14-93

**Published:** 2014-07-29

**Authors:** Mange Manyama, Jacinda R Larson, Denise K Liberton, Campbell Rolian, Francis J Smith, Emmanuel Kimwaga, Japhet Gilyoma, Kenneth D Lukowiak, Richard A Spritz, Benedikt Hallgrimsson

**Affiliations:** 1 Department of Anatomy, Catholic University of Health and Allied Sciences, P.O. Box 1464, Mwanza, Tanzania; 2 Department of Anatomy and Cell Biology, University of Calgary, Calgary, Canada; 3 Faculty of Veterinary Medicine, University of Calgary, Calgary, Canada; 4 Department of Surgery, Bugando Medical Centre, Mwanza, Tanzania; 5 Department of Physiology and Pharmacology, University of Calgary, Calgary, Canada; 6 Human Medical Genetics and Genomics Program, University of Colorado School of Medicine, Aurora, CO, USA

## Abstract

**Background:**

Orofacial clefts (cleft lip/palate; CL/P) are among the most common congenital anomalies, with prevalence that varies among different ethnic groups. Craniofacial shape differences between individuals with CL/P and healthy controls have been widely reported in non-African populations. Knowledge of craniofacial shape among individuals with non-syndromic CL/P in African populations will provide further understanding of the ethnic and phenotypic variation present in non-syndromic orofacial clefts.

**Methods:**

A descriptive cross-sectional study was carried out at Bugando Medical Centre, Tanzania, comparing individuals with unrepaired non-syndromic CL/P and normal individuals without orofacial clefts. Three-dimensional (3D) facial surfaces were captured using a non-invasive 3D camera. The corresponding 3D coordinates for 26 soft tissue landmarks were used to characterize facial shape. Facial shape variation within and between groups, based on Procrustes superimposed data, was studied using geometric morphometric methods.

**Results:**

Facial shape of children with cleft lip differed significantly from the control group, beyond the cleft itself. The CL/P group exhibited increased nasal and mouth width, increased interorbital distance, and more prognathic premaxillary region. Within the CL/P group, PCA showed that facial shape variation is associated with facial height, nasal cavity width, interorbital distance and midfacial prognathism. The isolated cleft lip (CL) and combined cleft lip and palate (CLP) groups did not differ significantly from one another (Procrustes distance = 0.0416, p = 0.50). Procrustes distance permutation tests within the CL/P group showed a significant shape difference between unilateral clefts and bilateral clefts (Procrustes distance = 0.0728, p = 0.0001). Our findings indicate the morphological variation is similar to those of studies of CL/P patients and their unaffected close relatives in non-African populations.

**Conclusion:**

The mean facial shape in African children with non-syndromic CL/P differs significantly from children without orofacial clefts. The main differences involve interorbital width, facial width and midface prognathism. The axes of facial shape differences we observed are similar to the patterns seen in Caucasian populations, despite apparent differences in cleft prevalence and cleft type distribution. Similar facial morphology in individuals with CL/P in African and Caucasian populations suggests a similar aetiology.

## Background

Orofacial clefts are the most common craniofacial birth defects, accounting for about 15% of all congenital abnormalities [1], with both genetic and environmental risk factors implicated in the underlying aetiology [2,3]. Normal variation of facial shape has also been reported to play a role in the familial transmission patterns of orofacial clefts [4,5].

Individuals with CL/P manifest craniofacial growth patterns different from those of their unaffected peers [6-8]. These differences could result from primary abnormalities of growth of facial and skull structures, as well as secondary influences of the cleft itself [9]. Facial shape differences that have been observed between individuals with CL/P and unaffected individuals include interocular distance or width, nasal base width, mouth width, lower facial height, nasal length and variable upper lip changes [6,8] and facial asymmetries in the orbital, nasal and maxillary regions [10,11]. Furthermore, somewhat atypical facial shape features have also been observed in parents and other close relatives of patients with CL/P, including thin upper lips, increased lower facial height and interorbital distances, and widening of the nasal cavity [4] as well as fluctuating and directional facial asymmetry [12]. These latter findings indicate that inherited predisposition to orofacial clefts includes phenotypic aspects of facial shape that are near the extremes of normal variation.

Worldwide, the prevalence of orofacial clefts varies based on geography and ethnicity [13,14]. Several studies have addressed facial shape variation associated with CL/P in Caucasian populations [6,7,15,16], in which the prevalence of clefts is 0.91 to 2.69 per 1000 live births [17,18]. However, there have been no reported studies of facial shape in individuals with CL/P in African populations, in which the prevalence of CL/P is considerably lower, 0.3 per 1,000 to 1.65 per 1,000 live births [19,20], and the proportions of cleft types is also somewhat different [21], perhaps reflecting different underlying genetic and environmental aetiologies.

Historically, approaches to evaluate facial shape in patients with CL/P have included traditional two-dimensional (2D) photography, direct anthropometry of facial structures, and soft tissue radiography [22-24]. These techniques suffer from measurement inaccuracy, lack of shape information from the un-captured third dimension, and possible radiation risk to the patient. Craniofacial growth changes take place in three dimensions (3D), and developmental changes of the soft tissues and underlying skeletal structures can thus be best analyzed using 3D imaging techniques, which provide more accurate quantitative shape information about craniofacial structures. Recent studies of CL/P have begun to utilize the 3D approach [6-8,15,16], but no study of 3D facial shape in CL/P has been reported in an African population. The aim of this study was to quantify 3D differences in facial shape between African (Tanzanian) children with unrepaired non-syndromic CL/P and unaffected control subjects using geometric morphometric techniques. Furthermore, as treatment of CL/P often occurs quite late in childhood in many African patients, usually without pre-surgical facial molding efforts, analysis of facial shape in Tanzanian children scheduled for CL/P reparative surgery prior to surgical correction offers a unique opportunity to study the progression of facial shape in children with untreated orofacial clefts.

## Methods

### Sample

All subjects were African, from northwestern Tanzania, medically assessed and treated at Bugando Medical Centre. The total sample was 126 children, 42 had unrepaired non-syndromic CL/P and 84 were unaffected controls. The CL/P were aged 2 to 120 months, while control group were aged 4 to 120 months. The mean age of the CL/P group was 29.4 ± 31.8 months and the mean age of the control group was 54.1 ± 34.5 months, the difference largely resulting from a greater number of infants in the CL/P group. Information on the structure of the sample in terms of sex, cleft status, and cleft type is shown in Table 1. We used opportunistic sampling for this study, given the location and population under study. We used all possible cases and for the controls, we doubled the sample size to better control for the effects of normal variation in facial shape across ages. Children with unrepaired non-syndromic CL/P were recruited consecutively as they attended surgical wards and clinics for the duration of the study. Children in the control group were recruited conveniently from Bugando Medical Centre and schools in the Mwanza region as long as they met the criteria below. None of the unaffected control children had known craniofacial anomalies and none had received orthodontic treatment. Further, none of the children with unrepaired orofacial clefts had undergone alveolar bone grafting or nasoalveolar molding (NAM) at the time of data collection. Ethical approval was granted from the Tanzania National Institute for Medical Research and the University of Calgary Conjoint Health Research Ethics Board (CHREB). Informed consent was obtained from the parents/guardians of all study subject children. All the facial figures are based on average shape (after morphing) of actual individuals in the unrepaired CL/P and Control groups.

**Table 1:** Distribution of individuals in the sample according to sex, cleft type and cleft laterality

**Parameter**	**n**	**CL/P**	**Control**	**Mean age ± SD**	***t*-test**	**p-value**
**Sex**						
Male	60	14	46	51.7 ± 36.4	1.212	0.228
Female	66	28	38	40.5 ± 34.1		
**Type of cleft**						
CL	34	34	-	30.7 ± 32.3		
CL/P	8	8	-	23.6 ± 31.2		
**Laterality**						
Right	10	10	-	30.3 ± 38.2		
Left	25	25	-	26.2 ± 30.9		
Bilateral	7	7	-	39.3 ± 29.2		

### Morphometric data

Data collection was done from 2008 to 2012. 3D facial surfaces were captured using the InSpeck 3D MegaCapturor white light digital stereophotogrammetric camera (Creaform Inc., Quebec, Canada), which acquires a 3D surface map in ~0.4 seconds using a 640 × 480 mm field of view with photo-realistic color and texture rendering. Facial scanning was performed with standardized head positions directly in front of the 3D camera while the subject was seated by themselves or on their mother’s lap, approximately 1 meter from the camera, with the lips at rest and eyes closed to minimize involuntary movement. Six standard views were obtained for each subject (frontal, left three-quarter profile, left, right three-quarter profile, right and inferior chin), which were processed and merged to obtain the full 3D facial image using InSpeck Facial Acquisition Processing Software (FAPS) and Editing and Merging software (EM) with a least possible error (approximately 0.300 mm) (Creaform Inc., Quebec, Canada).

**Figure 1:**
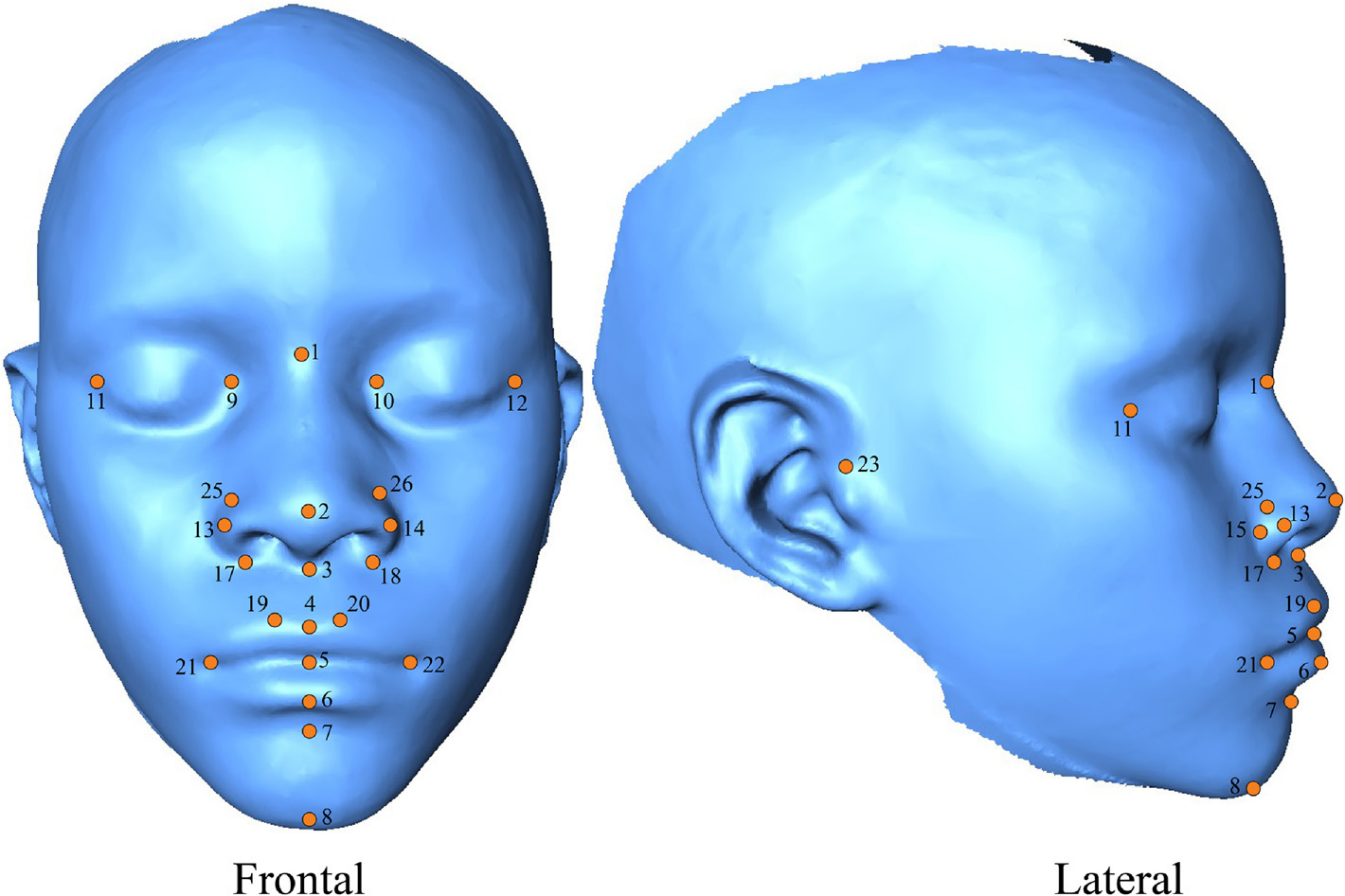
**Landmarked face.** 3D images (anterior and lateral views) showing 26 landmarks of interest around the face. Each landmark is numbered and corresponds to the landmarks in Table 1.

From the 3D reconstruction of each subject, 26 soft tissue facial landmarks (Figure 1 and Table 2) were used to characterize facial and nasolabial forms, chosen to provide coverage of soft-tissue facial structures [25,26]. The stomion landmark was taken as a midpoint of the labial fissure. However, when the lips were not closed in the rest position, it was taken as the midpoint of the lower border of the upper lip. The landmarks were recorded by one individual using MeshLab (V 1.3.0) [27], and the corresponding x,y,z coordinate locations were used for subsequent shape analysis. To ascertain landmark repeatability, a set of five control individuals was landmarked five times each, by one individual observer on five separate days. The average observation and variance was calculated for each landmark’s x,y and z dimensions. Across all landmarks and individual trials, observer precision was calculated to be 0.338 mm. This is well within the precision cited in other similar studies [28].

**Table 2:** List of landmarks used for evaluating facial shape

**Number landmark description**	
1 (M)	The saddle point of the sking corresponding to the intersection of the frontal and nasal bones.
2 (M)	Midline maximum of the curve of the nose; the nasal tip.
3 (M)	Junction of the nose and philtrum (base of the columella).
4 (M)	Midline of vermillion border of the superior lip between the christa philtri.
5 (M)	Midpoint of the labial fissure.
6 (M)	Midline of the vermillion border of the inferior lip.
7 (M)	The most posterior midpoint on the labiomental soft tissue contour between the lower lip and chin
8 (M)	Midline point on the inferior border of the mandible.
9 (R/L)	The inner corner of the eye where upper and lower eyelids meet.
11/12 (R/L)	The outer corner of eye where the upper and lower eyelids meet.
13/14 (R/L)	The most lateral point on the nasal alar crest.
15/16 (R/L)	The most posterolateral point at the junction between alar wing and face.
17/18 (R/L)	The edge of nasal ala where cartilage of the nose inserts the tissue above the upper lip.
19/20 (R/L)	The point at each crossing of the vermilion line and the elevated margin of the philtrum
21/22 (R/L)	Point marking the corner of the lips/mouth.
23/24 (R/L)	Tip of the eminence at the front of the ear.
25/26 (R/L)	The highest point of the junction between the alar wing and face.

### Shape analysis

MorphoJ v1.04a software was used to carry out geometric morphometric analysis of landmark configurations [29]. 3D landmark data for each individual were imported without object symmetry to preserve facial asymmetry resulting from unilateral clefts. The data were subjected to Procrustes superimposition to rescale to standard size, translate to standard position and rotate to standard orientation [30]. To compare unilateral versus bilateral clefts as a group, all individuals with right-side clefts were mirrored to have left-side clefts, by inverting the sign of the x-coordinate for all landmarks. Comparisons of left versus right, however, were carried out on the data prior to mirroring.

Standardization of the dataset for age and size was carried out in MorphoJ [29], using multivariate regression to create a line of best fit for age against the Procrustes coordinates and pooling the data by groups (control and cleft status). The 24 month difference in mean age between the controls and CL/P group is small enough that a linear approximation of shape change with age removes the vast majority of age-related shape variation. The only deviation from this occurs at the extreme lower end of CL/P group, where facial adiposity may introduce some uncontrolled variation into the sample; however, the slope of the age regressions look similar in both the control and CL/P groups. We then adjusted the age-standardized regression residuals for allometry by controlling for centroid size and these residuals, which are an adjusted set of 3D coordinates that describe facial shape after removing the age and size variation within each sample, were used in all subsequent shape analyses.

Using MorphoJ, facial shape variation was analyzed in the combined dataset of cleft and control children and also separately in the CL/P group (unilateral and bilateral) using Principal Component Analyses (PCA). Differences in shape between non-cleft children and unrepaired CL/P children and between unilateral and bilateral clefts in the unrepaired CL/P group were analyzed using Canonical Variate Analyses (CVA). Permutation analysis (10,000 permutations) for the mean Procrustes distance between groups as well as Procrustes ANOVA was used to assess significance of differences between groups. Since geometric morphometric techniques retain the intrinsic geometry in landmark coordinate data, shape variation along a given PC or CV axis can be displayed and visualized graphically. We compared groups using the set of age and centroid size regression residuals due to cleft status (cleft or control), cleft type (isolated CL or combined CL/P), bilateral versus unilateral, and cleft side (right versus left).

## Results

### Ontogeny, allometry and sexual dimorphism

**Figure 2:**
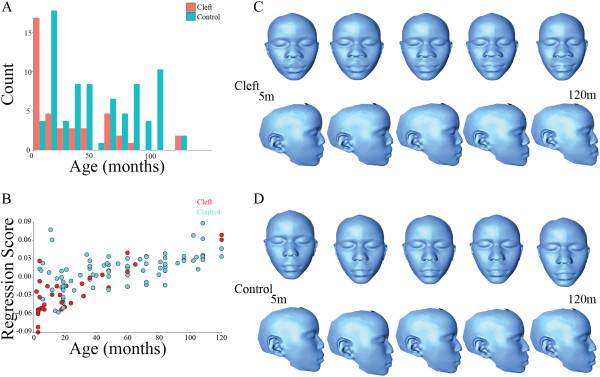
**Age variation in CL/P and controls. A**: A Histogram of age distribution for both controls and CL/P. **B**: Regressions of facial shape on age in CL/P and controls. **C**: Age morphs (thin plate spline warps) of individuals with CL/P sample means from 5 – 120 months. **D**: Age morphs (thin plate spline warps) of the control sample means from 5 – 120 months.

Pooled regression found age to explain 4.76% of the variation in the combined control and cleft sample and centroid size explains an additional 1.70% of the residual variation, indicating that facial shape does change with ontogeny and allometry and reinforcing the importance of controlling for age- and size- related facial variation within the two groups before further analysis (Figure 2). Procrustes analysis and other multivariate analyses were carried out after pooling the two groups together and regressing out the effects of age and centroid size on facial shape. Among the control group, the Procrustes distance permutation test showed that facial shape did not differ significantly between males and females (Procrustes distance = 0.0200, p = 0.1856). Among the CL/P group, the Procrustes distance permutation test showed that facial shape did not differ significantly between males and females (Procrustes distance = 0.0336, p = 0.5861). There is no evidence of sexual dimorphism in either group.

### Shape variation in the combined sample of CL/P and non-cleft children

**Figure 3:**
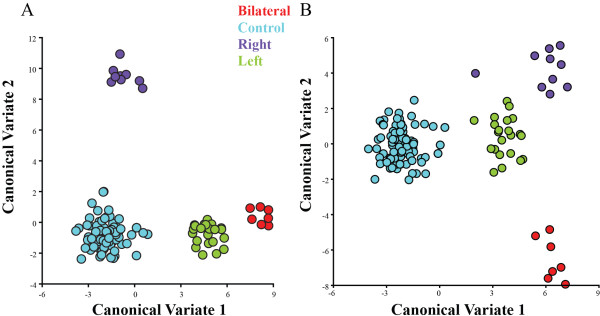
**Canonical Variate Analysis of individuals with CL/P and controls. A**: Plot of CVA scores for the control and CL/P groups showing separation by cleft side pre-mirroring along CV1 and CV2. **B**: Plot of CVA scores for the control and CL/P groups showing separation by cleft side post-mirroring along CV1 and CV2. The red data points represents individuals with bilateral CL/P, blue data points represents controls, purple data points represents individuals with right CL/P and green data points represents individuals with left CL/P.

**Figure 4:**
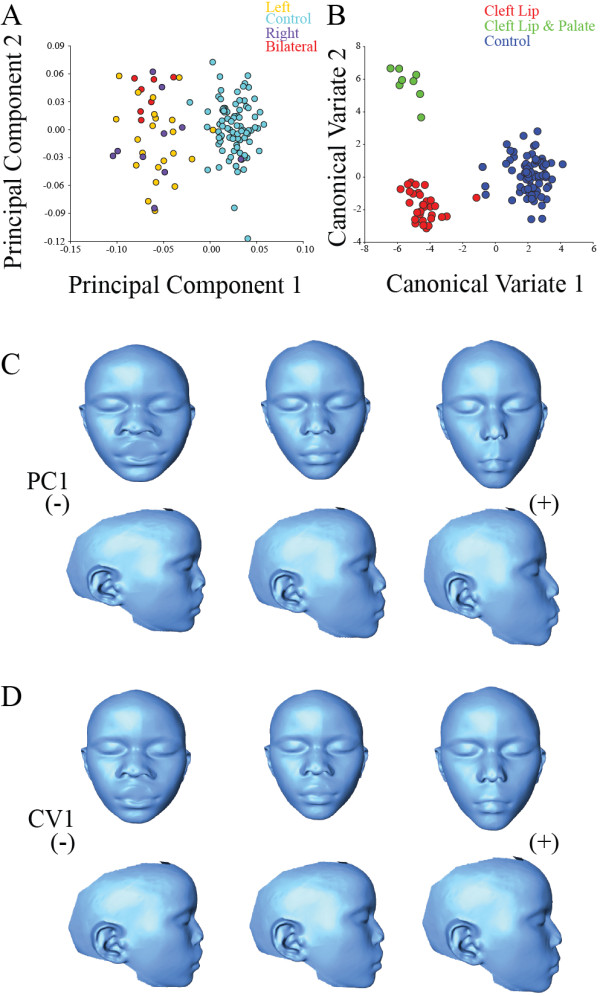
**Principal Component Analysis and Canonical Variate Analysis of individuals with CL/P and controls. A**: PCA of individuals within the control and CL/P showing separation of the groups along PC1 and PC2. **B**: Plot of CVA scores for the control and CL/P groups showing separation of the groups along CV1 and CV2. **C**: Morphs models of individuals within the control and CL/P showing extreme ends of the shape variation (in anterior and lateral views) associated with PC1. **D**: Morphs models of individuals within the control and CL/P showing extreme ends of the shape variation (in anterior and lateral views) associated with CV1.

First we performed a test for asymmetry due to cleft side (right versus left cleft) for individuals using Procrustes ANOVA on the pre-mirrored coordinates. We found a significant effect of directional asymmetry in the dataset (p < 0.0001), likely resulting from a larger number of individuals with left-sided clefts than right in the sample. A CVA plot showed clear separation due to cleft side pre-mirroring (Procrustes Distance = 0.0866, permutation p < 0.0001). Post-mirroring, the variance is reduced along both CV1 and CV2, with no significant mean shape difference between left- and right-sided clefts (Procrustes Distance = 0.0458, permutation p = 0.1787) (Figure 3). All further analysis were therefore based on the mirrored coordinates.On a PC plot, there is separation between the control group and CL/P individuals (Figure 4A). Within the CL/P group, there is significant variation within and among the different types of clefts. In this combined analysis, PC1 explains 19.41% of the variance and is associated with facial prognathism, nasal and mouth width and facial height (Figure 4C). PC2, which explains 11.98% of facial shape variance, is associated with interorbital distance, mandibular prognathism and flattening of the nose. PCs 3 and 4 explain 8.01% and 5.82% of the variance, respectively. Facial shape changes associated with PC3 included mainly maxillary and mandibular prognathism and, interorbital distance. PC4 is associated with nasal and interorbital distance.Separation of the isolated CL and combined CL/P groups can be seen in the CVA results (Figure 4B). CV1 showed that, compared to the control group, the CL/P group exhibits increased nasal and mouth width, increased interorbital distance, slightly prognathic premaxillary region and decreased facial height (Figure 4D). Permutation tests showed that Procrustes distances comparing the consensus facial shapes of the control versus CL groups (Procrustes distance = 0.0794) and control versus CL/P groups (Procrustes distance = 0.0878) were significantly different (p < 0.0001 for both), indicating that there is a difference in mean facial shape between these groups. The isolated CL and combined CL/P groups (Procrustes distance = 0.0416) were not significantly different (p = 0.50). Procrustes ANOVA in the combined sample (CL, CL/P and control individuals) supported the CVA results, in that cleft status contributed significantly (p < 0.0001) to overall shape variation in the sample.

### Facial shape variation within the CL/P group (unilateral and bilateral)

**Figure 5:**
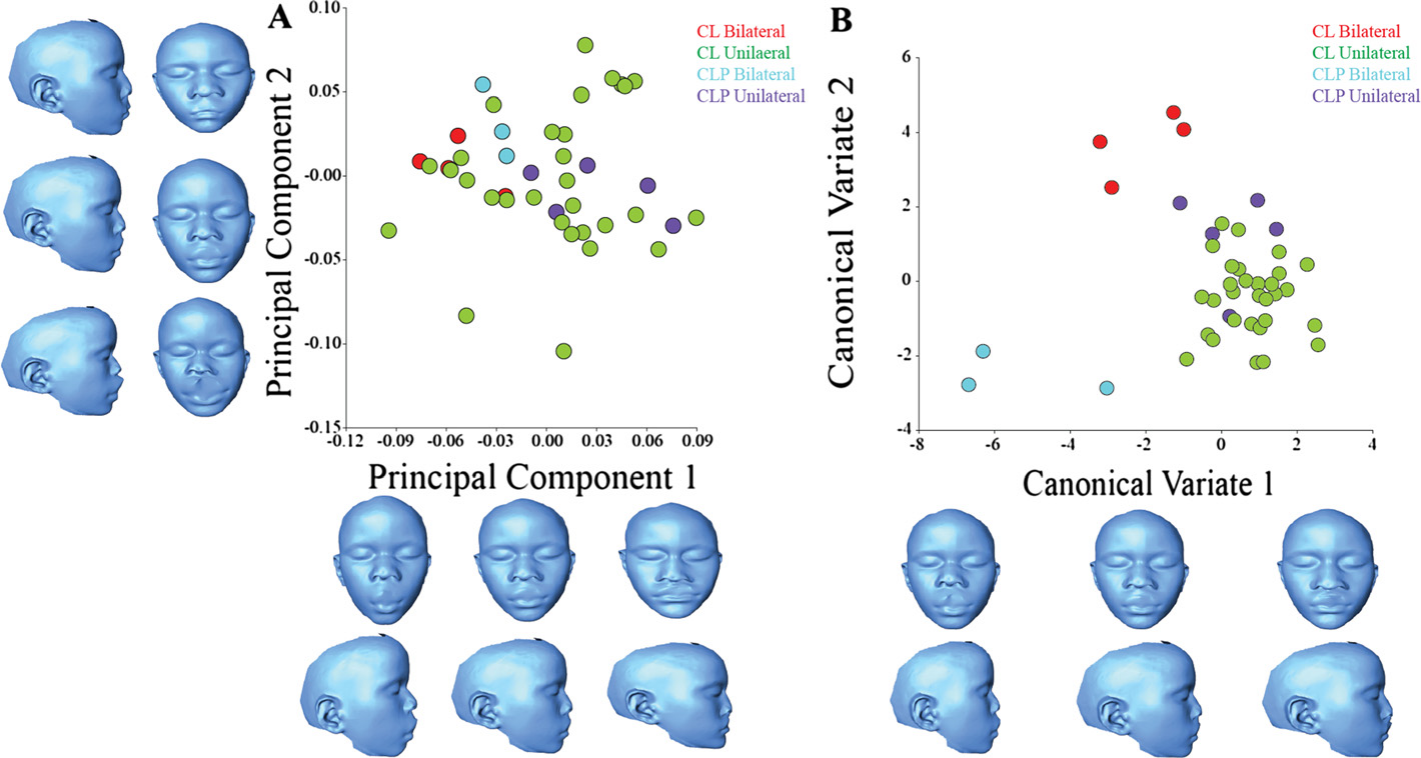
**Principal Component Analysis and Canonical Variate Analysis of individuals with CL/P. A**: PCA of individuals with CL/P showing PC1 and PC2 scores and their morphs. Morphs models shows extreme ends of the shape variation (in anterior and lateral views) associated with PC1 and PC2. **B**: Plot of CVA scores showing separation of individuals with CL/P along CV1 and CV2. Morphs models shows extreme ends of the shape variation (in anterior and lateral views) associated with CV1 and CV2. The red data points represents individuals with bilateral CL, green data points represents individuals with unilateral CL, blue data points represents individuals with bilateral CL/P, purple data points represents individuals with unilateral CL/P.

The first ten PCs accounted for 76% of total shape variance, with PC1 accounting for 17%. Variation along PC1 is associated primarily with lower nasal cavity width, lower facial height and mandibular and maxillary prognathism (Figure 5A). PC2 and PC3 account for approximately 12% and 10% of additional shape variance, respectively. PC2 is associated with interorbital distance, nasal width and maxillary prognathism. Shape changes associated with PC3 included mouth and nasal width and interorbital distance. PC4, which explained 8% of shape variance, described shape changes in nasal width, and positioning of the angle of the mouth.CVA showed some separation between cleft types, laterality and side (Figure 5B), although Procrustes distance permutation tests showed that facial shape did not vary between cleft types (isolated CL or combined CL/P) (Procrustes distance = 0.0416, p = 0.50), indicating there are no significant facial shape differences between individuals with isolated CL versus combined CL/P. Procrustes distance permutation tests within the CL/P group revealed a significant shape difference between unilateral clefts and bilateral clefts (Procrustes distance = 0.0728, p = 0.0001). Procrustes ANOVA of the CL/P individuals supported the permutation test results, showing that unilateral versus bilateral clefts were significantly (p = 0.0001) different in terms of shape variation in this sample. Comparison by cleft type (isolated CL versus combined CL/P) was again not significant (p = 0.79), indicating that combined cleft lip with cleft palate does not significantly differ in facial shape from isolated cleft lip in terms of surface facial morphology.

### Facial shape variation within the CL/P group (unilateral only)

Based on the significant contribution to facial shape variation by unilateral or bilateral clefts, we performed a separate analysis of the unilateral cleft group, in which we mirrored the left sides so that the clefts always appear on the “right” (N = 36). Permutation tests found no significant differences were seen between the cleft types (Procrustes distance = 0.0566, p = 0.25). In this analysis the first ten PCs explained 79.53% of facial shape variance in the group, with PC1 and PC2 accounting for approximately 17% and 14% of shape variance respectively. Variation along PC1 is associated primarily with nasal and mouth width, lower facial height and interorbital distance. PC2 is associated with nasal width, and maxillary prognathism.

## Discussion

Three-dimensional geometric morphometrics is a useful tool for assessing craniofacial form, providing detailed quantitative shape information about facial structures and regions. In this study, the facial shape of individuals with CL/P were analysed and compared with those from control subjects of the same ethnicity. This is the first study of facial shape differences in individuals with orofacial clefts in an African population.

Our results revealed various morphological differences in the CL/P group, none of which were sex-specific. Faces in the CL/P group were characterized by increased interorbital distance, decreased upper facial depth (in the anteroposterior direction), and prognathic premaxilla. These results are broadly consistent with similar studies in Caucasian CL/P patients and their relatives, which have reported increased facial and interorbital width [31-34], flattening of the face, prognathism of the premaxilla and mandible [33,35-39] and increased facial height [32,38,40].

The patterns of facial variation associated with orofacial clefts observed in this study could be a result of many factors, including genetic, environmental risk factors and their interactions. For example, variation in interorbital distance is associated with variation in midfacial outgrowth, which is regulated by *Shh* expression in the frontonasal ectodermal zone [41]. This dimension of facial shape could also be associated with variation in the rate of growth of the forebrain relative to the rate of outgrowth of the facial prominences, as suggested by animal studies [42]. This variable likely reflects an underlying developmental process, such as midfacial outgrowth or its relation to brain growth, that also plays an aetiologic role in CL/P in humans. Protrusion of the premaxilla, observed in the CL/P group, may relate to abnormal direction of alveolar growth and underdevelopment of the maxillary segments during early development [43]. Increased width of the embryonic midface might reflect improper or underdevelopment of embryonic facial prominences, thereby altering their spatial relationship and reducing the likelihood of correct contact and fusion [4]. This is supported by evidence from A/WySn mouse strains that have wider faces and increased risk of CL/P due to a *Wnt9b* hypomorphic allele [44,45]. Deficient or altered outgrowth of the maxillary prominences due to an *Ap2A* hypomorphic allele is also associated with CL/P in mice [46].

Flattening of the midfacial region in CL/P has been attributed to reduction in local growth of the medial nasal and maxillary prominences [33,44,47]. It has been argued that the rate of outgrowth of the facial prominences during face formation, particularly relative to growth of the brain, is associated with a variation in the window of opportunity for fusion of the facial prominences [45]. If variation in facial prominence outgrowth contributes to variation in facial prognathism, there might be a causal connection between the generally more prognathic faces seen in African populations [48] and the apparent lower incidence of CL/P in Africans.

Our results did not show significant facial shape differences between individuals with isolated CL versus patients with combined CL/P. This seems surprising, as combined CL/P is a more severe malformation that might be expected to result in greater global morphologic differences. Indeed, most previous studies of facial shape in CL/P have shown significant facial shape differences between these two groups [15,16,49]. The absence of apparent significant facial shape differences between the isolated CL and combined CL/P groups might be due to small sample size in the combined CL/P group in our study.

## Conclusions

This study has shown that facial shape variation associated with non-syndromic orofacial clefts in African children is similar to the patterns seen in Caucasian populations, despite apparent differences in cleft prevalence and cleft type distribution. Similar facial morphology in individuals with CL/P in African and Caucasian populations suggests that the developmental processes that are perturbed to produce clefts in these populations are generally similar. Differences in cleft frequency and type are thus not associated with qualitatively different patterns of facial shape variation in Tanzanian children from those previously reported in other populations.

## Competing interests

No financial or non-financial competing interests in this study.

## Authors’ contributions

MM made substantial contributions to the conception and design of this study, participated in data collection, statistical analysis and interpretation of results, drafted and revised the final manuscript. JRL contributed substantially to statistical analysis, draft of the manuscript and revised the final manuscript. DKL contributed substantially to the statistical analysis, draft and review of the manuscript. CR contributed substantially to the data collection, statistical analysis, draft of the manuscript and revised the final manuscript. FJS made substantial contributions to the data collection and processing. EK made substantial contributions to the data collection and review of the manuscript. JG operated most of individuals with orofacial clefts and contributed substantially to the data collection. KDL made substantial contributions to the data collection and review of the manuscript. RS made substantial contributions to the conception and design of this study and revised the final manuscript. BH made substantial contributions to the conception and design of this study, statistical analysis and draft of the manuscript. All authors have read and approved the final manuscript.

## Pre-publication history

The pre-publication history for this paper can be accessed here:

http://www.biomedcentral.com/1472-6831/14/93/prepub
